# Computational Analyses of the AtTPC1 (Arabidopsis Two-Pore Channel 1) Permeation Pathway

**DOI:** 10.3390/ijms221910345

**Published:** 2021-09-26

**Authors:** Carlos Navarro-Retamal, Stephan Schott-Verdugo, Holger Gohlke, Ingo Dreyer

**Affiliations:** 1 Centro de Bioinformática, Simulación y Modelado (CBSM), Facultad de Ingeniería, Campus Talca, Universidad de Talca, Talca 346000, Chile; 2 John von Neumann Institute for Computing (NIC), Forschungszentrum Jülich GmbH, 52425 Jülich, Germany; s.schott@fz-juelich.de; 3 Jülich Supercomputing Centre (JSC), Forschungszentrum Jülich GmbH, 52425 Jülich, Germany; 4 Institute of Biological Information Processing (IBI-7: Structural Bioinformatics), Forschungszentrum Jülich GmbH, 52425 Jülich, Germany; 5 Institute of Bio- and Geosciences (IBG-4: Bioinformatics), Forschungszentrum Jülich GmbH, 52425 Jülich, Germany; 6 Institute for Pharmaceutical and Medicinal Chemistry, Heinrich-Heine-Universität Düsseldorf, 40225 Düsseldorf, Germany

**Keywords:** TPC1, SV channel, permeability, molecular dynamics simulation, in silico electrophysiology

## Abstract

Two Pore Channels (TPCs) are cation-selective voltage- and ligand-gated ion channels in membranes of intracellular organelles of eukaryotic cells. In plants, the TPC1 subtype forms the slowly activating vacuolar (SV) channel, the most dominant ion channel in the vacuolar membrane. Controversial reports about the permeability properties of plant SV channels fueled speculations about the physiological roles of this channel type. TPC1 is thought to have high Ca^2+^ permeability, a conclusion derived from relative permeability analyses using the Goldman–Hodgkin–Katz (GHK) equation. Here, we investigated in computational analyses the properties of the permeation pathway of TPC1 from *Arabidopsis thaliana*. Using the crystal structure of AtTPC1, protein modeling, molecular dynamics (MD) simulations, and free energy calculations, we identified a free energy minimum for Ca^2+^, but not for K^+^, at the luminal side next to the selectivity filter. Residues D269 and E637 coordinate in particular Ca^2+^ as demonstrated in in silico mutagenesis experiments. Such a Ca^2+^-specific coordination site in the pore explains contradicting data for the relative Ca^2+^/K^+^ permeability and strongly suggests that the Ca^2+^ permeability of SV channels is largely overestimated from relative permeability analyses. This conclusion was further supported by in silico electrophysiological studies showing a remarkable permeation of K^+^ but not Ca^2+^ through the open channel.

## 1. Introduction

The Slowly activating Vacuolar (SV) channel was the first ion channel identified in plant vacuoles [1]. It is a voltage-gated, Ca^2+^-activated cation channel that activates upon membrane depolarization. For the model plant *Arabidopsis thaliana*, it was shown that the SV channel belongs to the class of Two Pore Channels (TPCs) and is encoded by the *TPC1* gene [2]. Cryo-EM and crystal structure analyses indicate that these channels are characterized by a tandem *Shaker* cassette structure that dimerizes via a C-terminal dimerization domain [3] to form a channel with 4 × 6 transmembrane helices [4,5]. In each *Shaker* cassette, the first four helices form a voltage-sensing domain (VSD), and helices 5 and 6 constitute the pore domain (P). The arrangement of the two subunits is domain-swapped, which means that the VSD1 of one Shaker cassette contacts P2 of the other cassette, while P1 contacts VSD2 of the same cassette. The four P-domains form the central permeation pathway.

TPCs are ubiquitously found in organelles of animals and plants and exist in three types, TPC1, 2, and 3, each with different gating and ion selectivity. They are ubiquitously found in organelles of animals and plants [6,7]. Nevertheless, plants have only TPC1-type channels that are equipped with EF hands in the linker between the two Shaker cassettes [8,9]. Plant TPC1s contribute to long-distance electrical and Ca^2+^ signaling and are essential players in vacuole excitability [10,11,12]. Patch-clamp experiments on plant vacuoles have indicated that the SV/TPC1 channel can conduct monovalent K^+^ and Na^+^ and divalent Ca^2+^ ions [6,13,14,15,16,17,18,19,20,21,22,23,24,25,26,27,28]. A standard procedure to determine the “permeability” of an ion channel is to adjust defined ion conditions on both sides of the membrane and then determine the voltage at which the current through the channel is zero. These zero-current voltage values are interpreted with the Goldman–Hodgkin–Katz equation [29], and permeability ratios are calculated. For the SV channel from *Vicia faba*, such analyses resulted in a permeability ratio of *P_Ca_:P_K_* ≈ 5:1 in one ionic condition [30] and *P_Ca_:P_K_* ≈ 1:10 in another [31]. The first value suggests that TPC1 is a Ca^2+^ channel with a slight K^+^ permeability, but the second implies the opposite. This led to the ongoing discussion of whether a significant amount of Ca^2+^ can pass the TPC1/SV channels under physiological conditions. By combining the patch-clamp technique with fura-2 fluorescence detection, electrical currents and changes in the Ca^2+^ concentration could be measured in parallel [32]. This approach indicated that the fractional calcium currents through TPC1 were approximately 10% of the total SV currents at elevated positive potentials.

To shed light on this puzzling set of partially contradicting reports about the permeability properties of TPC1, here we approached this topic from a structural and computational point of view. Using protein modeling, molecular dynamics (MD) simulations, and free energy calculations, we investigated the permeation patterns of different ions in AtTPC1 to elucidate which residues are involved in the translocation of ions and understand which structural features determine the selectivity of AtTPC1.

## 2. Results

### 2.1. Ions Modulate the Lateral Dimension of the Permeation Pathway

**Figure 1:**
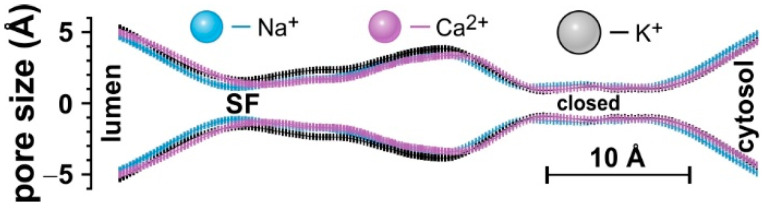
HOLE profile of the AtTPC1 pore in the presence of 0.15 M NaCl, 0.15 M KCl, or 0.1 M CaCl_2_. Data represent averages from five replica simulations with error bars indicating ± SEM; SF = selectivity filter. Please note that the used crystal structure shows AtTPC1 in the closed conformation. Therefore, the narrowest part of the permeation pathway at the cytosolic side is caused by the gate. For a rough orientation, Na^+^, K^+^, and Ca^2+^ are represented as spheres with ionic radii of Na^+^ ~ 1.1 Å, Ca^2+^ ~ 1.1 Å, and K^+^ ~ 1.5 Å [33]. The bar indicates the distance of 10 Å on the z-axis.

In the first set of simulations, we used the crystal structure of AtTPC1 in the closed state (PDB ID 5e1j) and embedded it in an explicit membrane of 1-palmitoyl-2-oleoyl-*sn*-glycero-3-phosphocholine (POPC). We established three different systems with salt concentrations of 0.15 M NaCl, 0.15 M KCl, or 0.1 M CaCl_2_. The systems were allowed to relax in a series of energy minimizations and heating simulations. Subsequently, 250 ns of molecular dynamics simulations were carried out for five independent replicas for each saline concentration. The pore and, in particular, the selectivity filter (SF) of AtTPC1 became markedly narrower in the presence of Na^+^ and Ca^2+^ compared to the system with K^+^ (Figure 1 and Figure S1). Hence, the permeation pathway of AtTPC1 did not have a static structure but interacted with the ions inside the pore and adjusted its geometry accordingly.

**Figure 2:**
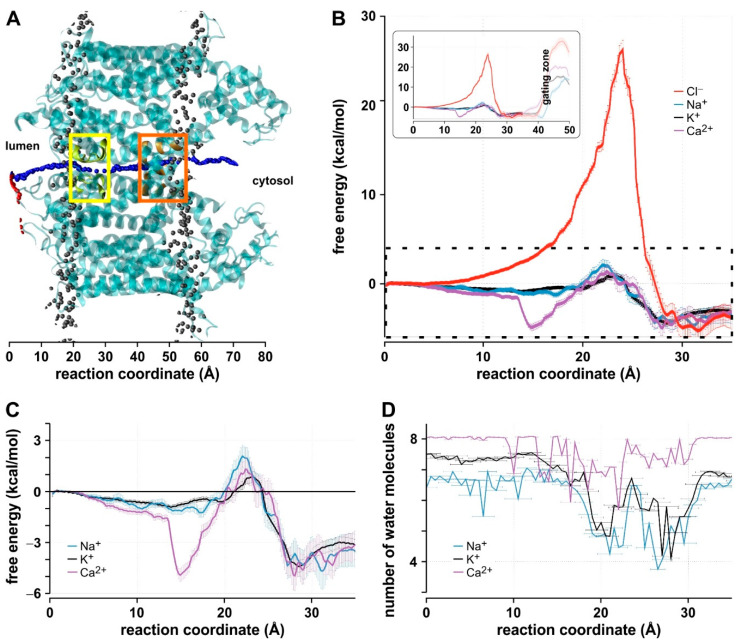
(**A**) Summary of the SMD protocol used to set up the US simulations. Red spheres indicate the first SMD simulations, orienting the ion at the luminal side of the channel. Blue spheres represent the second SMD step, pulling the ion across AtTPC1 through its pore. Phosphorus atoms are shown as gray spheres to indicate the location of the membrane. Yellow and orange boxes indicate the SF and the gating zone, respectively. (**B**) PMF profile of the ions Na^+^ (blue), K^+^ (black), Ca^2+^ (purple), and Cl^−^ (red) within the SF and central cavity of AtTPC1. The reaction coordinate indicates the position along the central cavity across the membrane up to the gating zone. The dashed box indicates the subsection displayed in (**C**). Inset: PMF profile along the entire pore. The gating zone is indicated. (**C**) Close-up of the dashed box in (**B**). (**D**) Number of water molecules surrounding the respective ion in the US simulations. Data are shown as mean ± SEM (*n* = 7).

Next, we computed the free energy profile of the different ions in the AtTPC1 permeation pathway. We performed steered molecular dynamics (SMD) simulations followed by umbrella sampling (US) simulations. For each salt condition, a single cation (Na^+^, K^+^, or Ca^2+^) was selected to go through the channel pore. As a negative control, Cl^−^ was taken from the KCl condition in an independent run. SMD simulations were carried out in two stages. First, the ion to be analyzed was aligned with the center of the SF at the luminal side in parallel to the membrane (Figure 2A, red spheres). Second, a force was applied to the ion to move it along the permeation pathway across the protein (Figure 2A, blue spheres). From this second simulation, we selected 100 frames as starting configurations for the subsequent US simulations. Starting from the initial condition, the next frame was chosen when the respective ion was at a distance of 0.5 Å with respect to its position in the previous frame. Thus, the 100 frames covered ion positions over a distance of 50 Å normal to the membrane plane. As the final 15 Å (30 frames) are dominated by the channel gate closing the permeation pathway [5], they were not further considered for the permeation analyses, focusing on the SF region instead (Figure 2B, inset).

From the umbrella sampling (US) simulations, the potentials of mean force (PMF) for the different ions at the remaining 70 positions along the pore were computed. The PMFs indicated significant differences between Cl^−^, Na^+^, K^+^, and Ca^2+^ (Figure 2B,C). Apart from Cl^−^, in the region of the selectivity filter (SF), the largest free energy barrier was 2.8 kcal·mol^−1^ for Ca^2+^, followed by 2.1 kcal·mol^−1^ for Na^+^, and 0.9 kcal·mol^−1^ for K^+^. Additionally, the energetic analyses pointed to a minimum in the PMF of −4.1 kcal·mol^−1^ for Ca^2+^ at the luminal side before the SF. Therefore, a Ca^2+^ ion permeating from the vacuolar lumen to the cytosol had to overcome an energy barrier of ~6.9 kcal·mol^−1^, whereas the largest barrier for a K^+^ ion (~1.4 kcal·mol^−1^) was only ~20% of this value. The largest barrier for Na^+^ (~3.3 kcal·mol^−1^) was about twice as high as the barrier for K^+^. Besides the PMF, the US simulations allowed us to estimate the number of water molecules surrounding the investigated ions (Figure 2D). At various locations along the permeation pathway, this number decreased. The decrease was greater for the monovalent K^+^ and Na^+^ than the divalent Ca^2+^. Nevertheless, at no place, the ions were fully dehydrated, indicating that the ions pass through the channel in a partially hydrated state as already suggested from crystal structure data [4,5]. It has been previously shown that Na^+^ has a higher hydration number than K^+^ [34,35] and that the dehydration energy is consequently higher for Na^+^ than for K^+^ [36]. Considering the free energy and hydration profiles, the analyses suggest that K^+^ permeation along the pathway is favored over Na^+^ and, in particular, Ca^2+^ permeation.

### 2.2. Residues Involved in the Ca^2+^ Affinity of the AtTPC1 Pore

**Figure 3:**
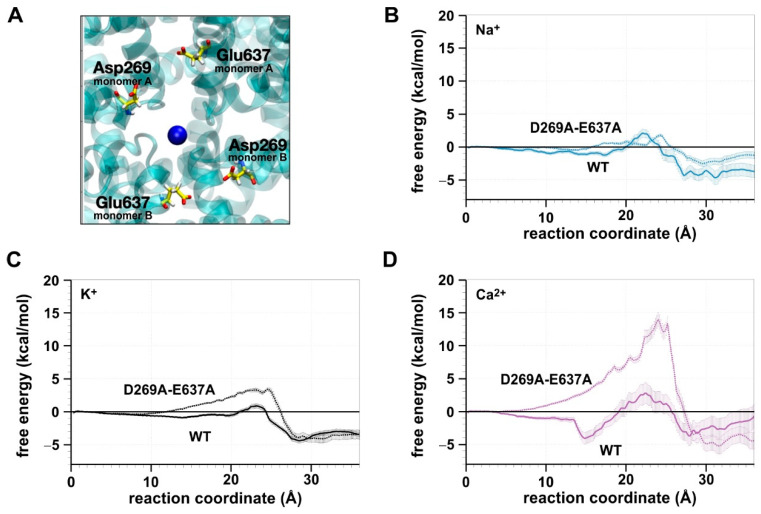
(**A**) Residues forming the presumable binding site for Ca^2+^ (blue): D269 and E637 from the two monomers (A and B) that form the channel. (**B**–**D**) PMF profiles of the ions Na^+^ (**B**), K^+^ (**C**), and Ca^2+^ (**D**). Comparison between the simulations with the AtTPC1 wildtype (continuous line) and the AtTPC1-D269A-E637A mutant (discontinuous, brighter line) is shown. Data are shown as mean ± SEM (*n* = 7).

To identify potential molecular causes for the high Ca^2+^ affinity in the AtTPC1 pore, we inspected the region at the luminal side before the SF. We identified four negatively charged residues (D269 and E637 in both monomers; Figure 3A) that could possibly act as a Ca^2+^ binding site. To assess the impact of these two sites on the permeation pathway, we created the in silico mutant AtTPC1-D269A-E637A, repeated the US simulations, and computed the PMFs (Figure 3B–D).

The mutations altered the free energy profile for all three ions. The largest effect, however, was observed for Ca^2+^ (Figure 3D). Removing the charges at positions 269 and 637 eliminated the minimum in the PMF at the luminal side before the SF and increased the highest energy barrier for Ca^2+^ to more than 10 kcal·mol^−1^. A sequence comparison of TPC1s from green algae, bryophytes, ferns, and angiosperms [9] indicates that, in particular, AtTPC1-D269 is highly conserved in the main TPC1-clade from land plants, while it is not in the bryophyte TPC1-b clade. By contrast, at the positions equivalent to AtTPC1-E637, there is a D/E/Q-polymorphism in both TPC1 clades (Figure S2). The negatively charged carboxy groups of the residues at these positions may facilitate the coordination of Ca^2+^ ions at the luminal entrance/exit of the permeation pathway of TPC1-type channels.

### 2.3. The AtTPC1 Pore Appears to Have a Preference for K^+^ over Na^+^ or Ca^2+^

**Figure 4:**
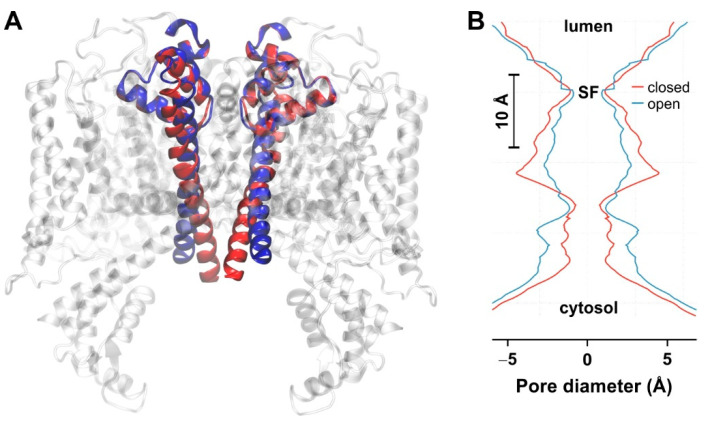
(**A**) Structural comparison between the closed (red) and open (blue) configuration of AtTPC1 shown in ribbon representation for TM I-S6 and II-S6; (**B**) HOLE profile of AtTPC1 in the presence of K^+^ in the closed (red) and open (blue) configuration. The bar indicates a distance of 10 Å along the z-axis.

For a more in-depth investigation of the ion permeation mechanism through AtTPC1, we constructed a homology model of this channel in the open configuration. We followed the protocol established by Guo et al. [5] to generate a chimeric model. As templates, we used the crystal structure of AtTPC1 in the closed configuration for all parts that are not directly involved in the gating process (~65% of the protein) and the crystal structure of the open NavMs sodium channel for those regions that primarily move during gating. The opening of AtTPC1 involves the lateral movement of the transmembrane helices IS6 and IIS6 (Figure 4A), as also found previously [5]. Initially, we analyzed the pore diameter and compared the open with the closed configuration of AtTPC1 (Figure 4B). Opening induced just a small change at the SF of about 0.3 Å, but at the cytosolic side the pore diameter widened by about 2 Å. This cross-bundle region has been described as the critical hydrophobic section that opens or closes the channel [5]. The widening observed in the gating region suggests that the generated model describes an open conformation of the channel. Based on this open-state model, we set up 30 different simulation sets; 15 for the AtTPC1 wildtype and 15 for the AtTPC1-D269A-E637A mutant, covering five replicas for each of the three salt concentrations (0.15 M KCl, 0.15 M NaCl, and 0.1 M CaCl_2_). Each system was equilibrated in MD simulations of 250 ns length.

Next, external fields with intensities of *E* = +0.180 kcal mol^−1^ Å^−1^ e^−1^ or *E* = −0.180 kcal mol^−1^ Å^−1^ e^−1^ were applied along the permeation pathway in the direction of the z-axis [37] to force a net ion flux in the two directions. These values are rather large and correspond to potential differences of ~±1 V across the membrane. They had to be chosen because TPC1 channels have a conductance of ~80 pS [38], which might allow for expecting a flux of 250 ions at ΔV ≈ 1 V in a computationally already very costly time interval of Δ*t* = 500 ns. For all six conditions, K^+^/+ΔV, K^+^/−ΔV, Na^+^/+ΔV, Na^+^/−ΔV, Ca^2+^/+ΔV, and Ca^2+^/−ΔV, we ran five independent simulations. In the time interval of Δ*t* = 500 ns, we counted from 0 to 100 K^+^ ions (A in Table 1; mean ± SD: 29 ± 30, median $\tilde{N}$ = 30). For Na^+^ and Ca^2+^, the numbers were much smaller (4 ± 6, $\tilde{N}$ = 1, and 1 ± 1, $\tilde{N}$ = 1, respectively), consistent with the calculated PMFs for these ions (Figure 2B), indicating that these ions permeated less well than K^+^ under the tested conditions. Although the maximal value of 100 for K^+^ is in the same order of magnitude as the expected value of 250, the failure to reach the theoretical value could be explained by saturation effects. At high voltage amplitudes, the current amplitude no longer follows a voltage increase linearly as assumed in the linear extrapolation of the experimental results. With respect to the current direction, we did not observe a significant difference. However, the dataset was too small to rule out such a difference. From the cytosol to the lumen (+ΔV), we counted 34 ± 39 K^+^ ions, while we registered 24 ± 22 K^+^ ions in the inverse direction from the lumen to the cytosol (−ΔV). When we repeated the forced ion flux simulations with the mutant AtTPC1-D269A-E637A, we observed that this mutation strongly reduced ion mobility for all three ions (B in Table 1). The data suggested that the flux from the lumen to the cytosol was more affected than the flow in the reverse direction. However, despite the enormous computational effort, the datasets were not large enough to allow for drawing statistically robust conclusions.

### 2.4. The Permeation Pathway of AtTPC1

**Figure 5:**
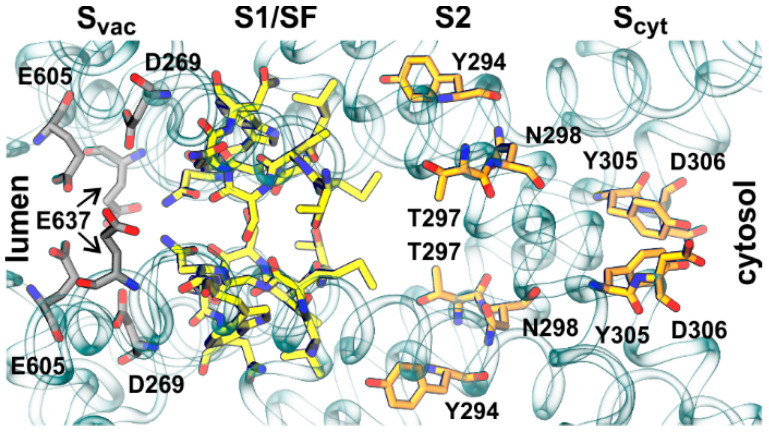
Ion permeation pathway of AtTPC1. Key sites/residues involved in the translocation of ions between vacuolar lumen and cytosol are highlighted: S_vac_ (grey), S1/SF (yellow), S2, and S_cyt_ (orange).

Ion flux simulations corroborated some predisposition of the AtTPC1 pore to K^+^ over Na^+^ or Ca^2+^. Therefore, we monitored in further detail the passage of a K^+^ ion along the permeation pathway and identified four sites that coordinated the movement: S_vac_ (D269, E637, and E605) and S1/SF closer to the luminal side, and S2 (Y294, N298, T297) and S_cyt_ (D306 and Y305) closer to the cytosolic side (Figure 5).

**Figure 6:**
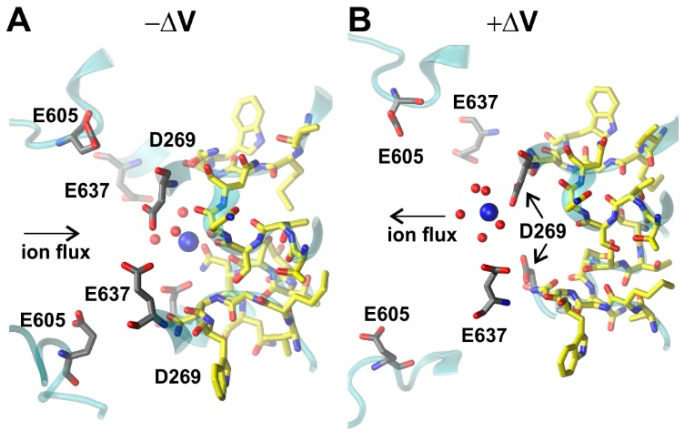
Structural comparison of the S_cyt_ (grey) and S1/SF (yellow) sites during efflux (**A**) and influx (**B**) simulations. K^+^ ions are displayed in blue; red spheres represent the oxygen atoms of water molecules. (**A**) During influx (−ΔV) simulations, E605 compacts towards D269/E637, which induces the translocation of K^+^ ions from the lumen toward the SF and, subsequently, facilitates their passage to the cytosol; (**B**) during efflux simulations (+ΔV), the D605/D606/E607 motif moves away from the S_vac_ site, facilitating K^+^ ions to exit the channel.

In contrast to K^+^ channels, ions are not entirely dehydrated while passing the SF of AtTPC1. Even in the SF region, four water molecules surround the K^+^ ion (Figure 2D). The closer inspection of the permeation pathway indicated that negative charges (S_vac_ and S_cyt_) as well as polar interactions (S1/SF and S2) dictated the passage of ions through the channel. When comparing +ΔV (K^+^ efflux) and −ΔV (K^+^ influx) simulations, we did not observe structural differences at positions S_cyt_, S2, and S1/SF. By contrast, two different configurations of S_vac_ were observed (Figure 6). S_vac_ comprises residue D269 together with E637, but also the highly flexible D605/D606/E607 motif located at the luminal side [4]. During influx (−ΔV) simulations, E605 compacted towards D269/E637 (Figure 6A), which induced the translocation of K^+^ ions from the lumen toward the SF and subsequently facilitated their passage to the cytosol. Additionally, during the simulations, we noticed that other ions accessed the vicinities of S_vac_ while the spotted ion tried to pass the SF. This observation suggested that the presence of these additional ions enhanced the frequency of successful attempts to pass the barrier of the SF due to charge repulsion. In contrast, during efflux simulations (+ΔV), we observed that the D605/D606/E607 motif moved away from the S_vac_ site (Figure 6B), facilitating K^+^ ions to exit the channel. In summary, these simulations further substantiated the conclusion of a dynamic and flexible nature of the permeation pathway.

## 3. Discussion

In electrophysiological experiments, TPC1-type channels have been attributed to permeation of a variety of monovalent and divalent cations [6,16,39]. The physiological significance of these apparent permeation properties has been debated for more than 25 years. In particular, a seemingly high Ca^2+^ permeability has since puzzled the field [14,26,30,31]. The crystal structures of AtTPC1 constituted a first breakthrough for putting this issue on proper grounds [4,5]. They allowed the conclusion that AtTPC1 has a multi-ion single-file pore [40], as do many other K^+^, Na^+^, and Ca^2+^ channels. Nevertheless, crystal structures are snapshots that provide only a static view of the channel. Therefore, we went one step further in this study and analyzed some dynamic properties of AtTPC1 by MD simulations and configurational free energy computations.

**Figure 7:**
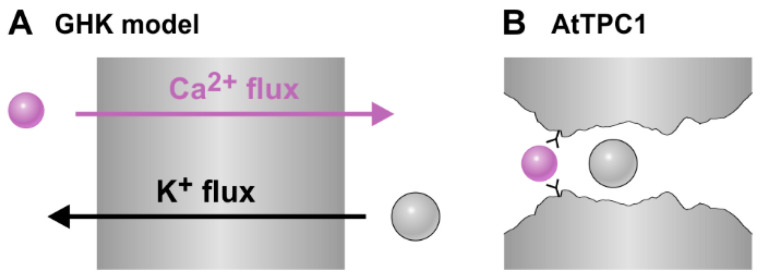
Difference between permeation according to the GHK model (**A**) and single-file permeation (**B**). (**A**) In the GHK model, the electrical current in one direction carried by Ca^2+^ ions is in a steady state neutralized by an inverse current carried by K^+^ ions flowing in the inverse direction. (**B**) In a single-file pore, a zero net current is established if the opposite fluxes block each other.

Our molecular dynamics simulations suggest that the pore structure of AtTPC1 is quite flexible and adapts to the permeating ion to some extent. The ions accommodate in the channel pore by maintaining part of their hydration shell. Although all three cations tested (Ca^2+^, Na^+^, and K^+^) fit well into the AtTPC1 pore, we were unable to provide substantial evidence that Ca^2+^ ions can permeate the channel better than K^+^. On the contrary, the pore seems to have a predisposition for K^+^ over Ca^2+^. This result appears to be contradictory to several experimental findings. AtTPC1 has been assigned a relative permeability sequence of Ca^2+^ > Na^+^∼Li^+^∼K^+^ > Rb^+^ > Cs^+^ [39]. This difference between model and experiment could theoretically be related to the fact that Ca^2+^ is difficult to handle in molecular simulations [41,42]. However, the supposed contradiction can be resolved in another way. For an explanation that reconciles modeling and experimental data, it is necessary to take a closer look at the basics of experimental permeability determination. In general, electrophysiological studies measure the voltage at which there is no net current flow across the membrane containing only—or almost exclusively—the type of channel under study. This value depends on the ion composition on both sides of the membrane. The set of voltage and concentration is then interpreted with the Goldman–Hodgkin–Katz (GHK) equation [29], and permeability ratios (“relative permeabilities”) are calculated. In this context, it should be noted that the GHK equation has been developed to describe the resting potential of excitable membranes as a whole and not of single channels [43]. It results from constant field theory and has the fundamental premise that the permeation of the various ions occurs independently (Figure 7A). In the GHK-model, the coupling between the different ion fluxes occurs solely via the membrane voltage. Thus, applying the GHK equation to poorly selective single-file ion channels such as AtTPC1 neglects the essential requirement of independent fluxes of the different ion species. In the single-file pore of AtTPC1, K^+^ and Ca^2+^, for instance, necessarily interact with each other (Figure 7B). Thus, for these channels, the model underlying the GHK equation is not valid. This also explains why different “relative permeability” coefficients result in different solution conditions [31].

The obtained “relative permeabilities” could therefore be strongly misleading, such as a P_Ca_/P_K_ ≈ 5:1 for TPC1s [30,39]. Such a value is obtained from the GHK equation when measuring AtTPC1 under bi-ionic conditions with 150 mM K^+^ (or Na^+^) at the cytosolic side and 15 mM Ca^2+^ at the luminal side. At 0 mV, the current is zero, which means that under this condition, the inwardly directed chemical Ca^2+^ gradient equals the outwardly directed K^+^ gradient (Figure 7A). If the membrane is a homogenous phase and K^+^ and Ca^2+^ do not interact with each other (prerequisites of the GHK equation), this would indeed mean that Ca^2+^ permeates better than K^+^. In such a condition, a ten-fold lower Ca^2+^ gradient would be sufficient to balance the flux induced by the K^+^ gradient. From the crystal structure and our MD simulations we conclude, however, that the permeation process through AtTPC1 is fundamentally different from that of the GHK model. When Ca^2+^ moves from the lumen to the cytosol and K^+^ from the cytosol to the lumen, both ions need to use the same pathway. In such a scenario, zero current means essentially no net flux at all (Figure 7B), while in the GHK model, zero current means that the macroscopic charge flux carried by Ca^2+^ in one direction is compensated by the charge flux carried by K^+^ in the other (Figure 7A). In other words, in the GHK model, the opposite fluxes compensate each other electrically, whereas in TPC1, the opposing Ca^2+^ and K^+^ block each other. The reason why just one tenth of the Ca^2+^ concentration is sufficient to block the K^+^ (Na^+^) flux at 0 mV [39] can be explained by a particularity of the AtTPC1 pore. Our analyses of the free energy profile of the permeation pathway (Figure 2) pointed to a highly conserved Ca^2+^ coordination site in plant TPC1s on the luminal side next to the selectivity filter. The identified site might not only have an influence on ion permeation but could also be responsible for ion concentration-dependent changes in the gating properties of SV channels [44]. The free energy minimum increases the probability of finding a Ca^2+^ ion at that position, which effectively has a concentrating effect. The vacuolar Ca^2+^ concentration used in the GHK equation is therefore a dramatic underestimate of the local surface Ca^2+^ concentration at the luminal entrance of the permeation pathway.

Our data can thus explain a “GHK-derived” relative permeability of P_Ca_/P_K_ > 1 even though Ca^2+^ permeates less favorably the AtTPC1 pore. This finding fits with the experimental observation of TPC1s having a higher conductance for K^+^/Na^+^ than for Ca^2+^ [6]. Qualitatively, our permeation simulations using an external electrical field are in agreement with these experimental data. K^+^ permeates markedly better than Ca^2+^ (Table 1). Nevertheless, we should also point to the obvious weaknesses of this particular simulation approach. In the absence of a crystal structure of the open AtTPC1, it had to be modeled in an open configuration introducing an initial degree of uncertainty. The subsequent application of an electrical field to the ions was equivalent to a transmembrane voltage of 1 V and, hence, about 10-fold larger than in electrophysiological experiments. This electrical field intensity was needed to observe about 100 ions crossing the selectivity filter during 500 ns of simulation time. Still, to obtain a statistically relevant number of permeation events, the sampling time would have to be increased by at least a factor of 100, which was beyond our reach. Nevertheless, given the current rise in computing power, such analyses might be achievable in the near future, which will also enable us to use membrane voltages in the physiological range for these types of simulations.

The simulation data presented in this study clarify that the relative permeabilities of SV channels/TPC1s calculated with the GHK equation are not very informative and do not say anything about the real permeability through these channels. Instead, they can be largely misleading by implying a high Ca^2+^ conductance from a relative permeability ratio of *P_Ca_:P_K_* ≈ 5:1. Under physiological conditions with ~100 mM K^+^ in the cytosol, 10–200 mM in the vacuole, submicromolar concentrations of Ca^2+^ in the cytosol and up to 1 mM in the vacuole, however, Ca^2+^ cannot be released by SV channels from the vacuole in significant amounts [45]. Our combined simulation data now provide further insights that can explain the apparent contradictions in the literature.

## 4. Materials and Methods

This work is based on the crystal structure of AtTPC1 (PDB ID 5e1j). The structure was prepared using the PRIME module of the Schrodinger suite of programs (Prime, version 3.9, Schrodinger, LLC, New York, NY, USA, 2017).

### 4.1. System Preparation

We embedded AtTPC1 in an explicit membrane of 1-palmitoyl-2-oleoyl-*sn*-glycero-3-phosphocholine (POPC) using the PACKMOL-Memgen module [46] of the Amber18 Molecular Dynamics Package [47,48]. To analyze the differences in affinity between the proteins and ions of interest, we established three different conditions by adding salt concentrations of 0.15 M NaCl, 0.15 M KCl, and 0.1 M CaCl_2_ for each protein system using the TIP3P water model [49]. The generated systems are comprised of ~300,000 atoms each. To prepare and relax the systems, a series of energy minimizations and heating simulations were performed using the Amber software package [47,50] with GPU acceleration [51], including a short NVT simulation for heating followed by a series of NPT simulations for density equilibration. After thermal stability simulations, 250 ns of production were performed. For each saline concentration, five independent replicas were performed. For all simulations, timesteps of 2fs were used. ff14SB and LIPID17 parameters were used to describe the protein and lipids, respectively. For the production runs, a Langevin thermostat was used, while the pressure was treated by a semi-isotropic pressure scaling. Regarding ions, the parameters obtained by Li et al. [42] were applied.

### 4.2. Free Energy Simulations

To properly analyze the differences in affinity between the tested ions and AtTPC1, Steered Molecular Dynamics (SMD) simulations followed by Umbrella Sampling (US) simulations were performed. For each salt condition, a single cation (Na^+^, K^+^, or Ca^2+^) was selected to go through the channel pore. Additionally, Cl^−^ was taken from the KCl condition as a negative control in an independent run. Two runs of SMD simulations were performed. In the first run, a force with a constant of 1.0 kcal·mol^−1^·Å^−2^ was applied to the ion for 0.01 ns, aligning it with the center of mass (COM) of the SF of AtTPC1 in the xy plane (membrane plane), while maintaining a distance of ~26 Å in the z-axis, which is perpendicular to the membrane plane. To run multiple single axis restraints, sander was used. In the following, a second pulling simulation was performed, applying a force with a constant of 1.0 kcal·mol^−1^·Å^−2^ for the monovalent ions and 1.5 kcal·mol^−1^·Å^−2^ for the divalent ions, to move the ion across the protein normal to the membrane plane, placing it at a distance of ~50 Å from the starting point below the SF on the cytosolic side during 0.130 ns. From the latter simulation, 100 frames in which the ion was at a distance of 0.5 Å with respect to each other were selected as starting conformations for the US simulations. During the US simulations, the distance to the SF in the z-axis was used as a reaction coordinate, using a harmonic potential with a force constant of 10 kcal·mol^−1^·Å^−2^ to maintain the ion position. In order to avoid lateral movements of the ion and to ensure a proper sampling in the space surrounding the pore of the channels, we used a flat-bottom restraint in the xy-plane with radius 8 Å with respect to the COM of the SF and a harmonic restraint with a force constant of 10 kcal·mol^−1^·Å^−2^. To prevent other ions from accessing the SF, two distance restraints with respect to the center of mass of the SF and to the residues forming the cross region were applied. For both, a force constant of 5 kcal·mol^−1^·Å^−2^ was used and a minimum distance of 15 Å from each reference point was enforced. For each window, 10 ns of MD simulation were performed, from which the first 3 ns were discarded as equilibration, and the remaining 7 ns were used in order to generate the PMF. To estimate the unbiased free energy profile for the ion permeation, the WHAM algorithm was used [52] with a total of 300 bins and a tolerance of 10^−7^. In each PMF, the first and last 10 points are considered in the bulk water and used to shift the profile. The error was estimated from the PMF of every nanosecond of the production runs. The width of the permeation pathway was monitored with the program HOLE [53].

### 4.3. MD Simulations in the Presence of an External Electrical Field

To investigate the ion permeation mechanism through TPC, an open configuration of AtTPC1 was constructed. A homology model was built using the crystal structure of an open NavMs sodium channel (PDB ID 5hvx) as an additional template. We followed the strategy described by Guo et al. [5] to obtain an open AtTPC1 model using the PRIME module of the Schrodinger program suite (Prime, version 3.9, Schrodinger, LLC, New York, NY, 2017). Considering that only IIS4 has been shown to contribute to voltage-gating but not IS4 [5,54], we created a chimeric model as follows: we used as a template the closed state of AtTPC1 (PDB ID 5e1j) to model IS1–IS6 plus both EF hands (EF1–EF2) (Gln32-His416), and to model the pore region between IIS5–IIS6 of the second domain. Thus, ~65% of the whole model was based on the crystal structure of the closed state of AtTPC1 (100% of identity between the modeled and the template sequence). For the remaining parts, i.e., from IIS1 to IIS6 (Ser417-Lys686), with the exception of the pore region, we used the NavM crystal structure (PDB ID 5hvx) as a template to induce channel opening. In this region, the sequence identity between modeled and template sequence was ~24.6%. The comparison of both structures (closed vs. open configuration of AtTPC1), revealed an RMSD of 2.1 Å over the entire protein, but only 0.5 Å RMSD in the region of the SF, indicating that the selectivity filters of both structures were almost identical. The models were additionally evaluated by using TopScore [55], which provides a meta quality assessment of the protein structure at the global and per-residue level by estimating the uncertainty in a scale from 0 to 1 (Figure S3). To evaluate the permeability of the different channels and understand the structural features responsible for the different permeation profiles, regular molecular dynamics (MD) simulations were performed applying an external electrical field at different saline conditions. After minimizations and thermal equilibrations, five independent MD simulations of 250 ns were performed at different salt concentrations (0.15 M KCl, 0.15 M NaCl, and 0.1 M CaCl_2_). Subsequently, external fields with intensities of *E* = ±0.180 kcal·mol^−1^·Å^−1^·e^−1^ were applied in the direction of the z-axis to induce inward and outward currents, respectively. All ion permeations events were measured by an in-house script.

## Figures and Tables

**Table 1:** Simulation of ion permeation through (**A**) AtTPC1 and (**B**) AtTPC1-D269A-E637A forced by an electrical field. Positive (+ΔV) and negative (−ΔV) electrical fields were applied along the permeation pathway, and the number of ions passing the selectivity filter was counted in a time interval of 500 ns (*n* = 5 for each condition). The upper values indicate mean ± SD, the middle values the range between minimum and maximum number of observations, and the lower values the median. Simulations were performed with salt concentrations of 150 mM K, 150 mM NaCl, and 100 mM CaCl_2_, respectively.

A	AtTPC1		
Mean ± SD	Total Flux	Cytosol → Lumen +ΔV	Lumen → Cytosol −ΔV
Range [min…max]			
$\tilde{N}$ Median			
K^+^	29 ± 30	34 ± 39	24 ± 22
	[0…100]	[4…100]	[0…46]
	$\tilde{N}$ = 30	$\tilde{N}$ = 29	$\tilde{N}$ = 30
Na^+^	4 ± 6	6 ± 8	2 ± 3
	[0…20]	[1…20]	[0…6]
	$\tilde{N}$ = 1	$\tilde{N}$ = 1	$\tilde{N}$ = 1
Ca^2+^	1 ± 1	1 ± 1	1 ± 1
	[0…3]	[1…3]	[0…2]
	$\tilde{N}$ = 1	$\tilde{N}$ = 1	$\tilde{N}$ = 0
**B**	**AtTPC1-D269A-E637A**		
K^+^	7 ± 14	13 ± 19	1 ± 1
	[0…46]	[0…46]	[0…2]
	$\tilde{N}$ = 1	$\tilde{N}$ = 7	$\tilde{N}$ = 0
Na^+^	1 ± 2	3 ± 2	0 ± 0
	[0…6]	[0…6]	[0…0]
	$\tilde{N}$ = 0	$\tilde{N}$ = 2	$\tilde{N}$ = 0
Ca^2+^	0 ± 0	0 ± 0	0 ± 0
	[0…1]	[0…1]	[0…0]
	$\tilde{N}$ = 0	$\tilde{N}$ = 0	$\tilde{N}$ = 0

## Data Availability

All data that support the findings described in this study are available within the manuscript or the related supplementary information. Additional information is available from the corresponding authors upon reasonable request.

## Supplementary Materials

The following are available online at https://www.mdpi.com/article/10.3390/ijms221910345/s1. References are included here in the reference list [9,55].
