# Supplementary material for: Computational Analyses of the AtTPC1 (Arabidopsis Two-Pore Channel 1) Permeation Pathway
Source: Int J Mol Sci. 2021 Sep 26;22(19):10345. doi: 10.3390/ijms221910345 (PMC8508871; doi:10.3390/ijms221910345)
Supplement: Supplementary file 1 [file ijms-22-10345-s001.zip › ijms-1349711-supplementary.pdf]

Supplementary Figure S1

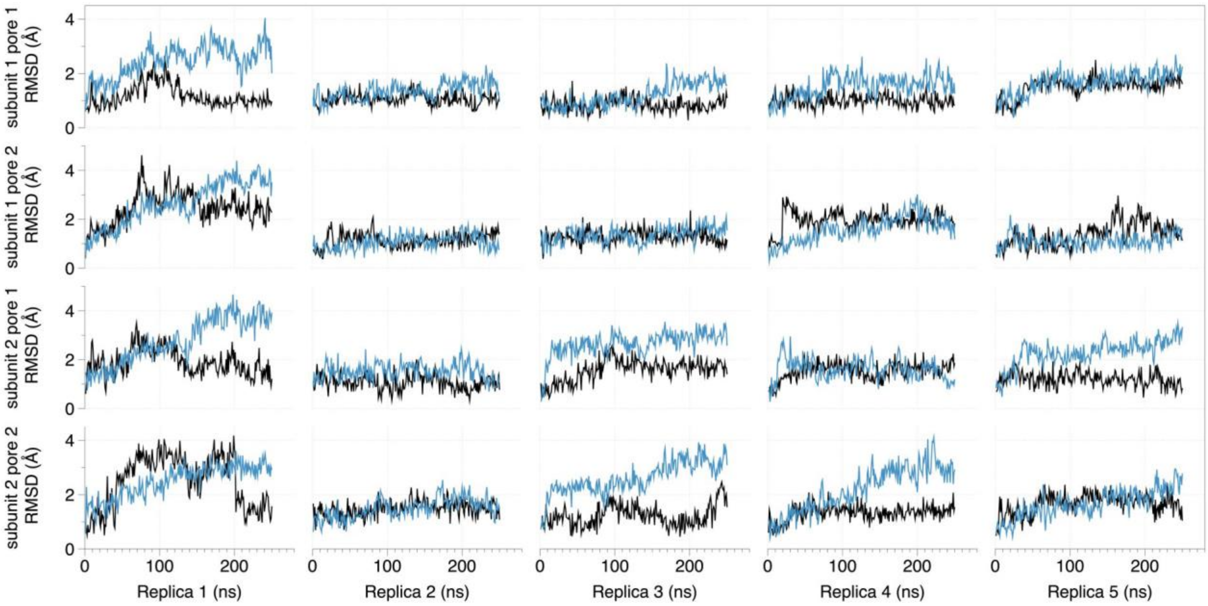

Figure S1. Structural stability of the selectivity filter. Analysis of root-mean-square deviation (RMSD) of the backbone of residues forming the selectivity filter of AtTPC1 in the presence of 0.15 M KCl (black) and 0.15 M NaCl (blue).

Supplementary Figure S2

	AtTPC1-D269		AtTPC1-E637	
Medicago_truncatula_Medtr2g090380.1	NLILAVVYDSFKSELV	[...]	GVWEELMESYKDLT	Angiosperm TPC1
Arabidopsis_thaliana_AT4G03560.1	NLILAVVYDSFKEQLA	[...]	GNWQVWMESYKDLT	
Vitis_vinifera_GSVIVT01020549001	NLILAVIYDSFKNQLA	[...]	NNWQVWMQSYKDLT	
Chenopodium_quinoa_LOC110703516	NLILAVVYDSFKSELA	[...]	GNWQAWMQTYWDLT	
Chenopodium_quinoa_LOC110685197	NLILAVVYDSFKSELA	[...]	GNWQAWMQTYWDLT	
Populus_trichocarpa_Potri.013G131100.2	NLVLAVVYDSFKDQLV	[...]	GNWHEWMQSYKDLT	
Nicotiana_tabacum_Nitab4.5-0000512g0020.1	NLILAVVYDSFKSELV	[...]	GNWQVWMQSYKELT	
Aquilegia_coerulea_Aqcoe4G103300.1	NLILAVVYDSFKEQLV	[...]	GNWQVWMEGYKELT	
Spirodela_polyrhiza_Spipo14G0028300	NLILAVVYDSFKAQLA	[...]	GNWQVWMESYRDLT	
Oryza_sativa_LOC_Os01g48680.1	NLILAVIYDSFKEQLA	[...]	GNWQAWMESYRQLT	
Salvinia_cucullata_Sacu-v1.1-s0093.g019069	NLVLAVVYDSFKDQLA	[...]	GNWQVWLESFVILT	Fern TPC1
Blechnum_spicant_scaffold-VITX-2006239	NLILAVVYDSFKEQLA	[...]	GNWQVWLESYVTLT	
Thuidium_delicatulum_scaffold-EEMJ-2039009	NLVLAVVYDSFKGQLA	[...]	GNWHVWMDGFLEVT	Bryophyte TPC1a
Physcomitrella_patens_Pp3c15-12230V3.1	NLVLSVVYDSFKGQLA	[...]	GNWHIWMDGFQEVT	
Sphagnum_fallax_Sphfalx04G006400.1	NLVLAVVYDSFKGQLA	[...]	GNWQVWMDGYAQLT	
Takakia_lepidozioides_scaffold-SKQD-2009335	NLVLAVVYDSFKGQLA	[...]	SNWHVFMDGYAELT	
Physcomitrella_patens_Pp3c1-6272V3.1	NLVLAVVYDSFKGQLA	[...]	GSWQTWMDSYVVLT	
Physcomitrella_patens_Pp3c1-6270V3.1	NLVLAVVYDSFKGQLA	[...]	GSWQTWMDSYVVLT	
Thuidium_delicatulum_scaffold-EEMJ-2007306	NLILAVVYDSFKGQLA	[...]	GNWQSWMDSFVILT	
Marchantia_polymorpha_Mapoly0002s0274.1	NLVLAVVYDSFKGQLA	[...]	GNWQVWMETYGILS	
Nothoceros_vincentianus_scaffold-TCBC-2005470	NLILAVVYNSFKEQFA	[...]	GNWQIWMESYAHLT	
Klebsormidium_nitens_kfl00312-0080-v1.1	NLVLAVIFDSFQKQLG	[...]	ADWQDWMEGYAILG	Klebsormidium TPC1
Klebsormidium_nitens_kfl00110-0290-v1.1	NLTLAVIFDSFKNQLG	[...]	ASWQDWMEGYAVLS	
Klebsormidium_nitens_kfl00097-0180-v1.1	SLILAVVYNGYKDQLT	[...]	TLYSDWQSIASNIY	
Klebsormidium_nitens_kfl00001-0210-v1.1	SLIFAVVYDGYKNSLA	[...]	SLFSVWLTIAGRIY	
Klebsormidium_nitens_kfl00012-0290-v1.1	NLMLAVIYEAFKEQVV	[...]	SSWLTYQDAFVRAT	
Chara_australis_CL6562.Contig1-All.p1	SLLFSVVFRGYRQRLA	[...]	RDWIVFAKGYVAAT	Chara TPC1
Marchantia_polymorpha_Mapoly0050s0061.1	HLLFALIYNNYKVQLA	[...]	NNWYVIMDGIVAAS	Bryophyte TPC1b
Marchantia_polymorpha_Mapoly0062s0036.1	NVAFAVIYTNFKRQMA	[...]	GNWYIIMEGYSAAT	
Physcomitrella_patens_Pp3c3-16950V3.1	NLVFTVIYSNYKAQMV	[...]	NNWYVFMDAYAVAT	
Takakia_lepidozioides_scaffold-SKQD-2009261	NLVFSVIYSNYKAQIA	[...]	NNWYVFMDAYAAAT	
Sphagnum_fallax_Sphfalx16G086000.1	NLAFMVIYSNYKVEIA	[...]	NNWYVIMDAYAFVT	
Sphagnum_fallax_Sphfalx19G080400.1	NFAFTVIYSSYKAQIA	[...]	NNWYVIMDAYAIVT	
Sphagnum_fallax_Sphfalx03G070700.1	NLAFSVIYSNYKAQMA	[...]	NKWYVFMDAYAAVT	
Buxbaumia_aphylla_scaffold-HRWG-2017281	NLAFSVIYSSYKAQMA	[...]	NKWYVFMEAYAAVT	
Physcomitrella_patens_Pp3c19-18680V3.1	NLAFSLIYSNYKAQMA	[...]	NKWYVFMDAYAAVT	
Thuidium_delicatulum_scaffold-EEMJ-2039845	NLAFSVIYSSYKQQMA	[...]	NKWYVFMDAYAAVT	
Physcomitrella_patens_Pp3c18-10190V3.1	NLAFSVIYSSYKAQMA	[...]	NKWYVFMDAYAAVT	
Physcomitrella_patens_Pp3c21-11410V3.1	NLAFSVIYSSYKAQMA	[...]	NKWYVIMDAYAAVT	
Takakia_lepidozioides_scaffold-SKQD-2080274	NLVFSVIYSNYKAQMA	[...]	NKWYVIMDAYAVAT	
Sphagnum_fallax_Sphfalx16G017900.1	SLAFSVVYSNYKSQMV	[...]	NKWYVIMDAYAAAT	
Buxbaumia_aphylla_scaffold-HRWG-2013254	NLAFSVIYSNYKAQMA	[...]	NKWYVIMDGFAAAA	
Physcomitrella_patens_Pp3c14-20770V3.1	NLAFSVVYSNYKTQMA	[...]	NKWYVIMDGYAAAT	
Physcomitrella_patens_Pp3c1-28370V3.1	NLAFSVVYSNYKAQMA	[...]	NKWYVIMDGYAAAT	
Physcomitrella_patens_Pp3c2-10230V3.1	NLAFSVIYSNYKAQMA	[...]	NKWYVIMDGYAAAT	

Figure S2. Sequence comparison of TPC1s from different species. Shown are the regions around AtTPC1-D269 and AtTPC1-E637. Sequence data are from [9].

Supplementary Figure S3

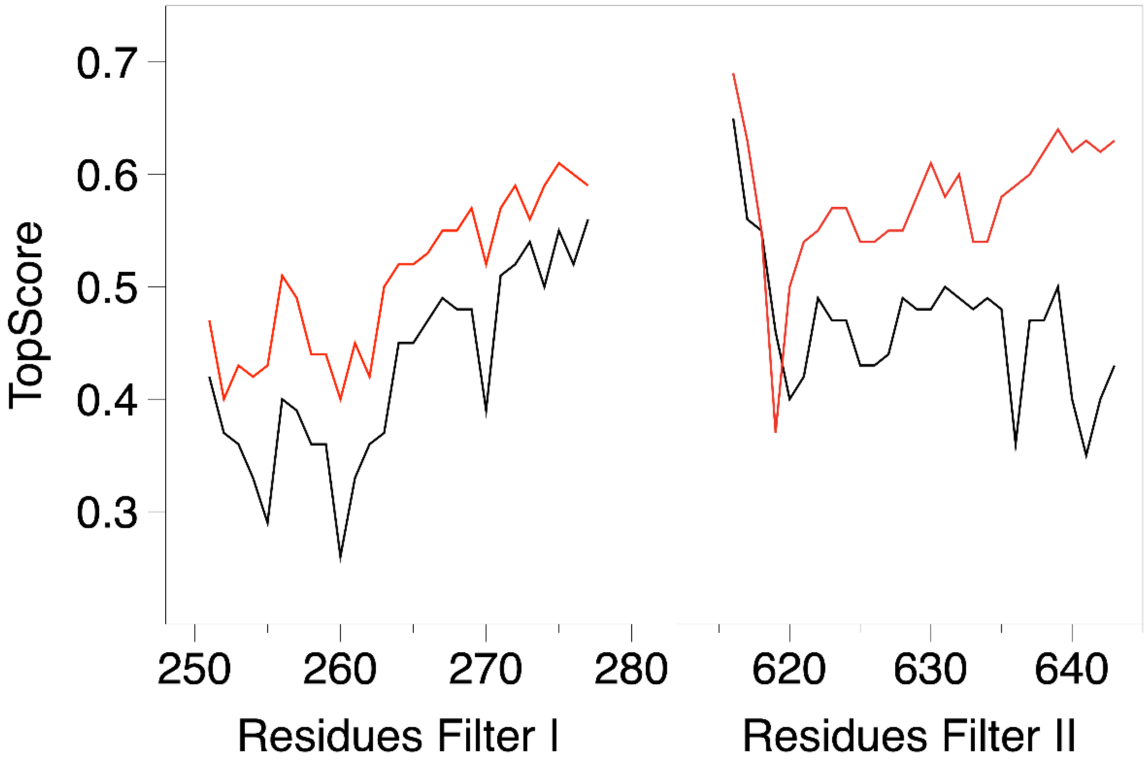

Figure S3. Per-residue TopScore of the filter regions in the AtTPC1 crystal structure of the closed configuration (black) and in the model of the open configuration (red). TopScore [55] provides a meta quality assessment of the protein structure at the global and per-residue level by estimating the error in a scale from 0 to 1. There is only a moderate increase in the estimated error between the crystal closed configuration and the modelled open configuration. The largest differences are observed for the selectivity filter residues in the second domain of AtTPC1. These residues are located in the vicinity of the IIS6 helix, which is the helix responsible for channel opening.
